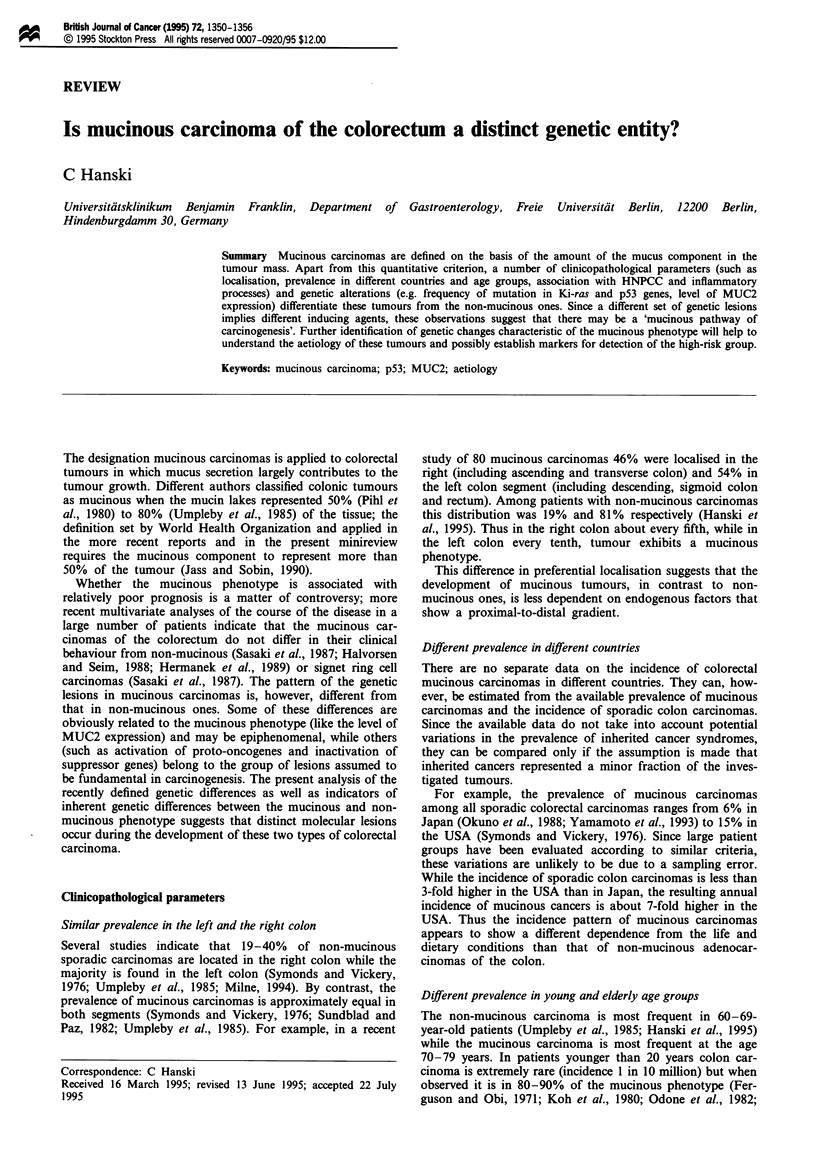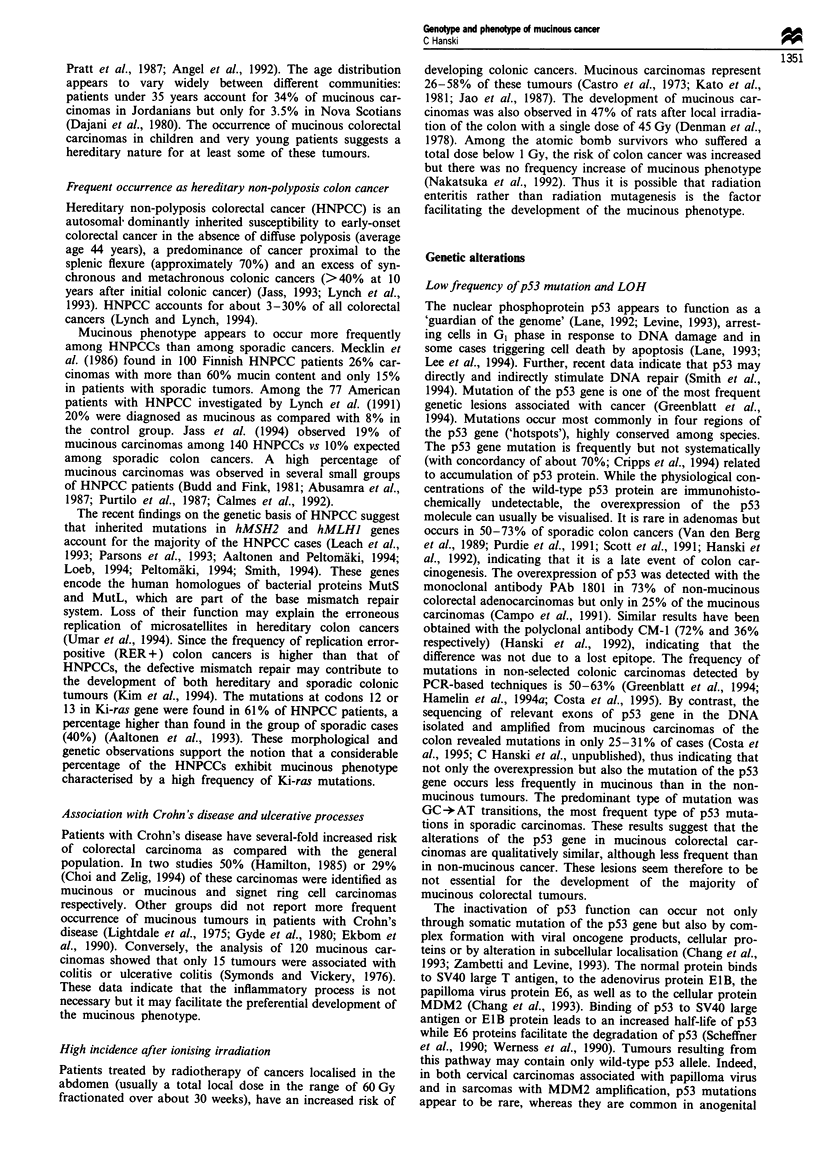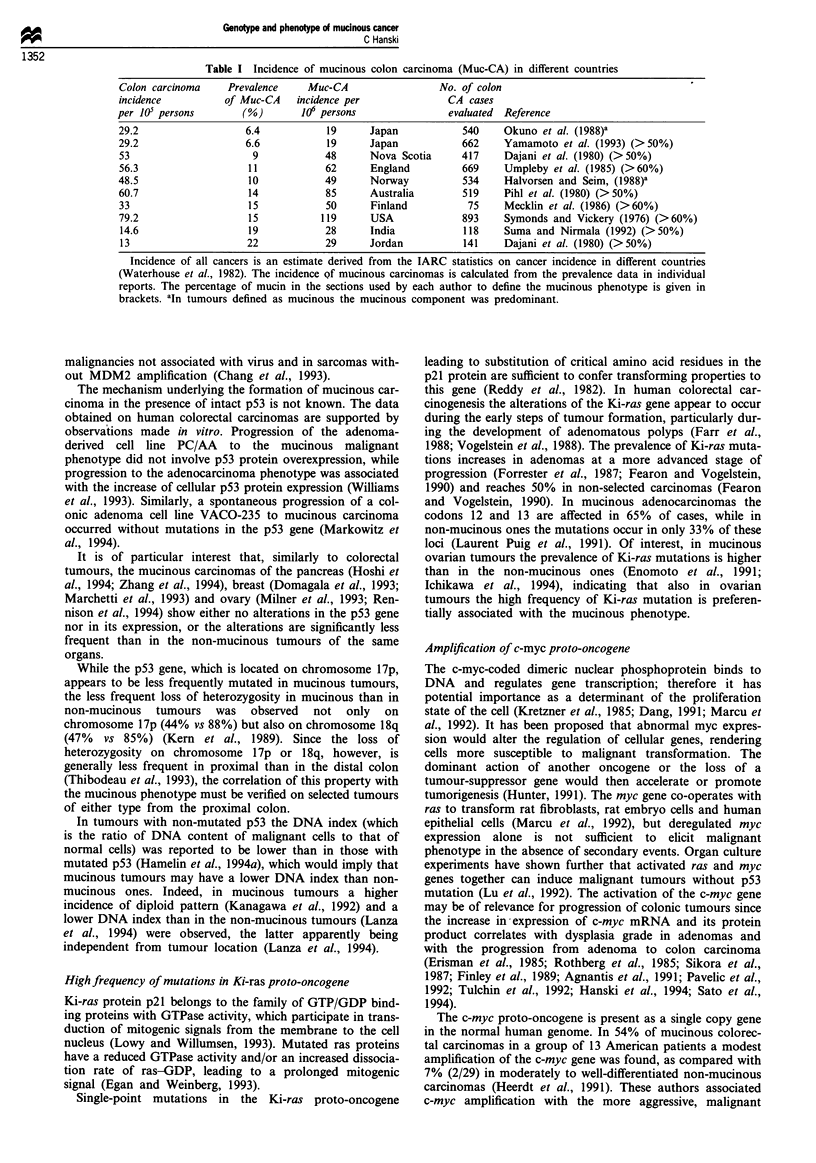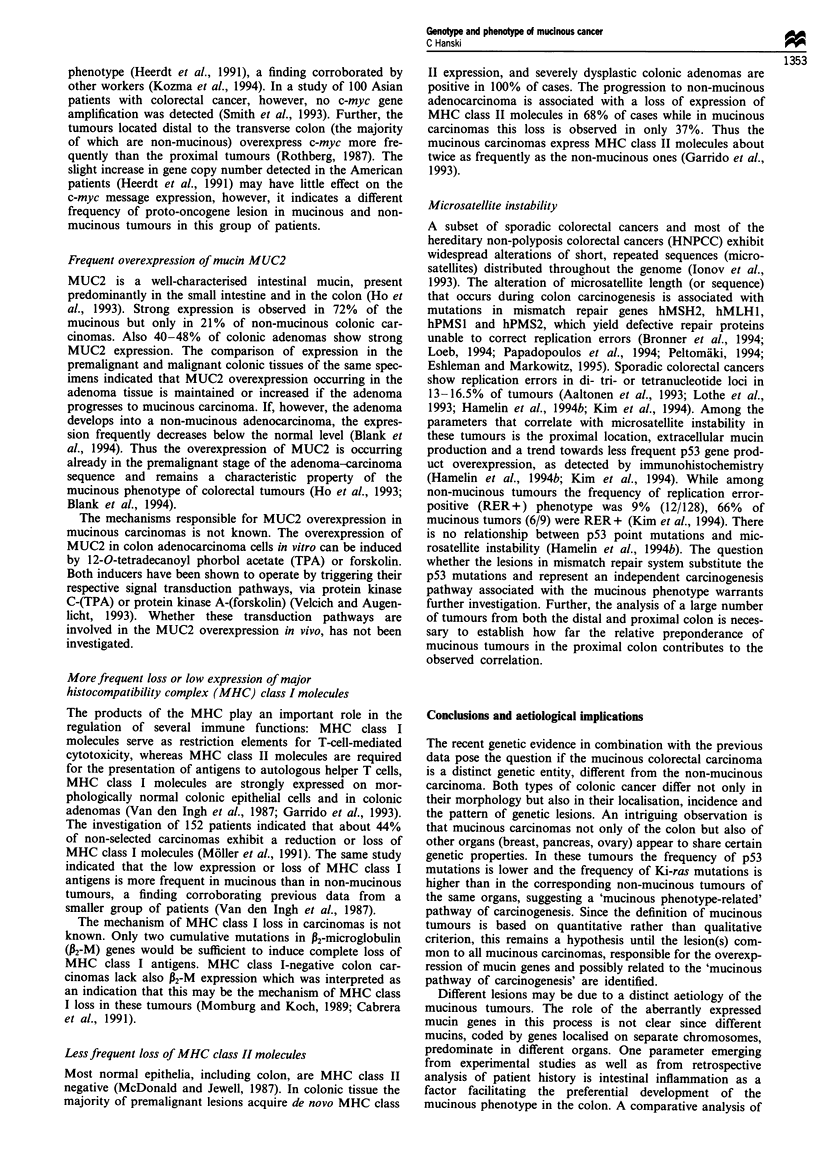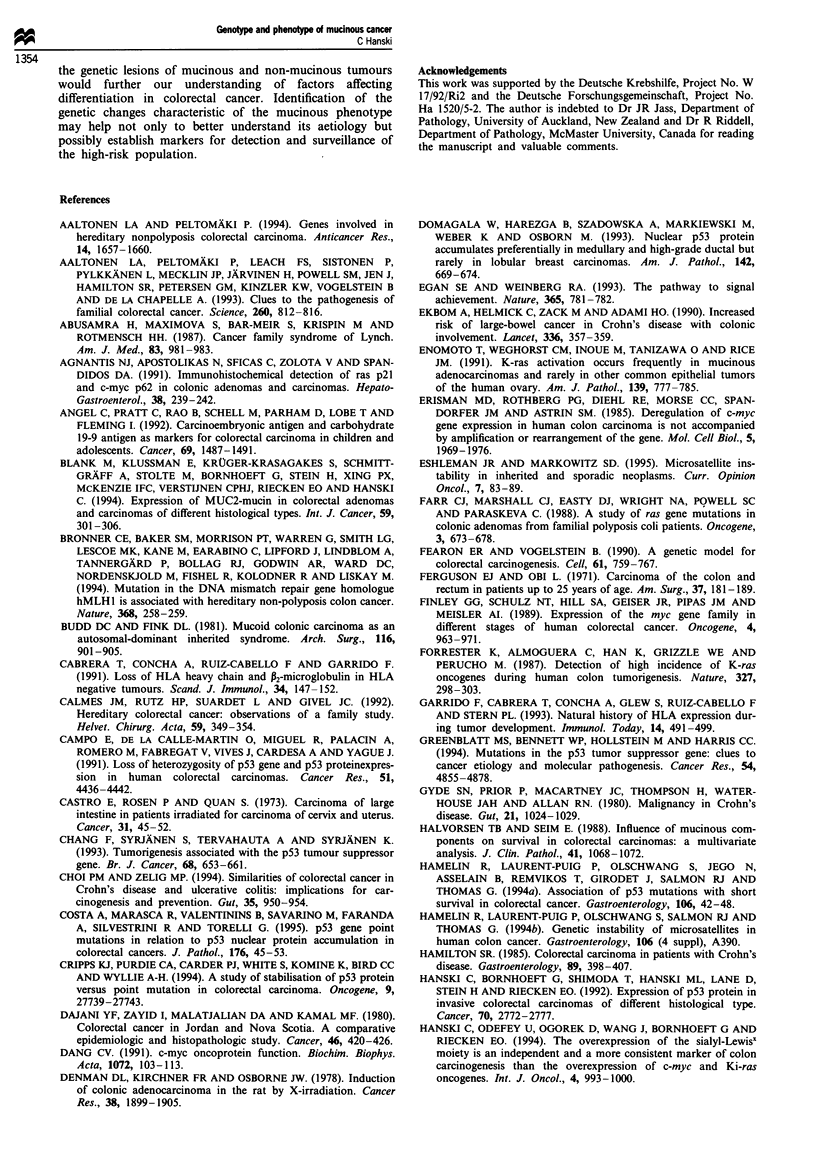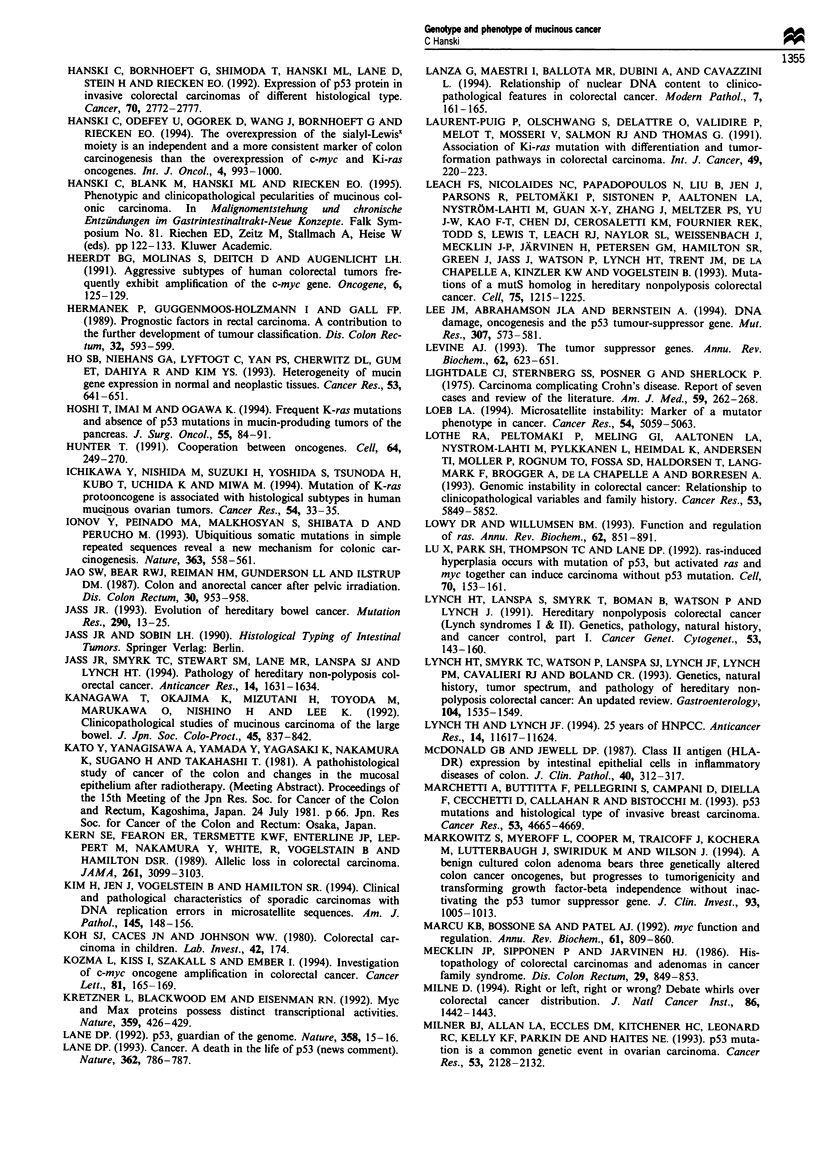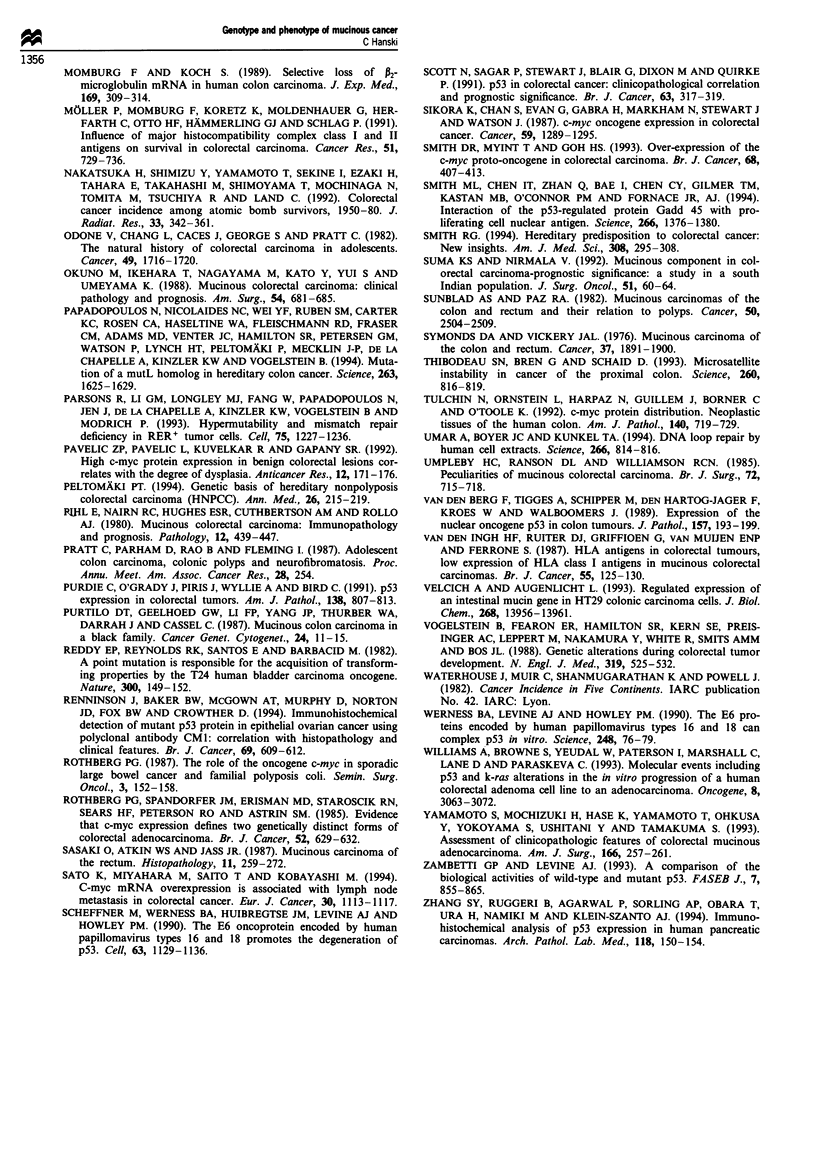# Is mucinous carcinoma of the colorectum a distinct genetic entity?

**DOI:** 10.1038/bjc.1995.514

**Published:** 1995-12

**Authors:** C. Hanski

**Affiliations:** Universitätsklinikum Benjamin Franklin, Department of Gastroenterology, Freie Universität Berlin, Germany.

## Abstract

Mucinous carcinomas are defined on the basis of the amount of the mucus component in the tumour mass. Apart from this quantitative criterion, a number of clinicopathological parameters (such as localisation, prevalence in different countries and age groups, association with HNPCC and inflammatory processes) and genetic alterations (e.g. frequency of mutation in Ki-ras and p53 genes, level of MUC2 expression) differentiate these tumours from the non-mucinous ones. Since a different set of genetic lesions implies different inducing agents, these observations suggest that there may be a 'mucinous pathway of carcinogenesis'. Further identification of genetic changes characteristic of the mucinous phenotype will help to understand the aetiology of these tumours and possibly establish markers for detection of the high-risk group.


British Journal of Cancer (1995) 72, 1350-1356
© 1995 Stockton Press All rights reserved 0007-0920/95 $12.00

REVIEW

### Is mucinous carcinoma of the colorectum a distinct genetic entity?

C Hanski

*Universitätsklinikum Benjamin Franklin, Department of Gastroenterology, Freie Universität Berlin, 12200 Berlin, Hindenburgdamm 30, Germany*

**Summary** Mucinous carcinomas are defined on the basis of the amount of the mucus component in the tumour mass. Apart from this quantitative criterion, a number of clinicopathological parameters (such as localisation, prevalence in different countries and age groups, association with HNPCC and inflammatory processes) and genetic alterations (e.g. frequency of mutation in Ki-*ras* and p53 genes, level of MUC2 expression) differentiate these tumours from the non-mucinous ones. Since a different set of genetic lesions implies different inducing agents, these observations suggest that there may be a 'mucinous pathway of carcinogenesis'. Further identification of genetic changes characteristic of the mucinous phenotype will help to understand the aetiology of these tumours and possibly establish markers for detection of the high-risk group.

**Keywords:** mucinous carcinoma; p53; MUC2; aetiology

The designation mucinous carcinomas is applied to colorectal tumours in which mucus secretion largely contributes to the tumour growth. Different authors classified colonic tumours as mucinous when the mucin lakes represented 50% (Pihl *et al.*, 1980) to 80% (Umpleby *et al.*, 1985) of the tissue; the definition set by World Health Organization and applied in the more recent reports and in the present minireview requires the mucinous component to represent more than 50% of the tumour (Jass and Sobin, 1990).

Whether the mucinous phenotype is associated with relatively poor prognosis is a matter of controversy; more recent multivariate analyses of the course of the disease in a large number of patients indicate that the mucinous carcinomas of the colorectum do not differ in their clinical behaviour from non-mucinous (Sasaki *et al.*, 1987; Halvorsen and Seim, 1988; Hermanek *et al.*, 1989) or signet ring cell carcinomas (Sasaki *et al.*, 1987). The pattern of the genetic lesions in mucinous carcinomas is, however, different from that in non-mucinous ones. Some of these differences are obviously related to the mucinous phenotype (like the level of MUC2 expression) and may be epiphenomenal, while others (such as activation of proto-oncogenes and inactivation of suppressor genes) belong to the group of lesions assumed to be fundamental in carcinogenesis. The present analysis of the recently defined genetic differences as well as indicators of inherent genetic differences between the mucinous and non-mucinous phenotype suggests that distinct molecular lesions occur during the development of these two types of colorectal carcinoma.

### Clinicopathological parameters

### *Similar prevalence in the left and the right colon*

Several studies indicate that 19–40% of non-mucinous sporadic carcinomas are located in the right colon while the majority is found in the left colon (Symonds and Vickery, 1976; Umpleby *et al.*, 1985; Milne, 1994). By contrast, the prevalence of mucinous carcinomas is approximately equal in both segments (Symonds and Vickery, 1976; Sundblad and Paz, 1982; Umpleby *et al.*, 1985). For example, in a recent study of 80 mucinous carcinomas 46% were localised in the right (including ascending and transverse colon) and 54% in the left colon segment (including descending, sigmoid colon and rectum). Among patients with non-mucinous carcinomas this distribution was 19% and 81% respectively (Hanski *et al.*, 1995). Thus in the right colon about every fifth, while in the left colon every tenth, tumour exhibits a mucinous phenotype.

This difference in preferential localisation suggests that the development of mucinous tumours, in contrast to non-mucinous ones, is less dependent on endogenous factors that show a proximal-to-distal gradient.

### *Different prevalence in different countries*

There are no separate data on the incidence of colorectal mucinous carcinomas in different countries. They can, however, be estimated from the available prevalence of mucinous carcinomas and the incidence of sporadic colon carcinomas. Since the available data do not take into account potential variations in the prevalence of inherited cancer syndromes, they can be compared only if the assumption is made that inherited cancers represented a minor fraction of the investigated tumours.

For example, the prevalence of mucinous carcinomas among all sporadic colorectal carcinomas ranges from 6% in Japan (Okuno *et al.*, 1988; Yamamoto *et al.*, 1993) to 15% in the USA (Symonds and Vickery, 1976). Since large patient groups have been evaluated according to similar criteria, these variations are unlikely to be due to a sampling error. While the incidence of sporadic colon carcinomas is less than 3-fold higher in the USA than in Japan, the resulting annual incidence of mucinous cancers is about 7-fold higher in the USA. Thus the incidence pattern of mucinous carcinomas appears to show a different dependence from the life and dietary conditions than that of non-mucinous adenocarcinomas of the colon.

### *Different prevalence in young and elderly age groups*

The non-mucinous carcinoma is most frequent in 60–69-year-old patients (Umpleby *et al.*, 1985; Hanski *et al.*, 1995) while the mucinous carcinoma is most frequent at the age 70–79 years. In patients younger than 20 years colon carcinoma is extremely rare (incidence 1 in 10 million) but when observed it is in 80–90% of the mucinous phenotype (Ferguson and Obi, 1971; Koh *et al.*, 1980; Odone *et al.*, 1982;

Correspondence: C Hanski
Received 16 March 1995; revised 13 June 1995; accepted 22 July 1995

Pratt *et al.*, 1987; Angel *et al.*, 1992). The age distribution appears to vary widely between different communities: patients under 35 years account for 34% of mucinous carcinomas in Jordanians but only for 3.5% in Nova Scotians (Dajani *et al.*, 1980). The occurrence of mucinous colorectal carcinomas in children and very young patients suggests a hereditary nature for at least some of these tumours.

### *Frequent occurrence as hereditary non-polyposis colon cancer*

Hereditary non-polyposis colorectal cancer (HNPCC) is an autosomal dominantly inherited susceptibility to early-onset colorectal cancer in the absence of diffuse polyposis (average age 44 years), a predominance of cancer proximal to the splenic flexure (approximately 70%) and an excess of synchronous and metachronous colonic cancers (>40% at 10 years after initial colonic cancer) (Jass, 1993; Lynch *et al.*, 1993). HNPCC accounts for about 3–30% of all colorectal cancers (Lynch and Lynch, 1994).

Mucinous phenotype appears to occur more frequently among HNPCCs than among sporadic cancers. Mecklin *et al.* (1986) found in 100 Finnish HNPCC patients 26% carcinomas with more than 60% mucin content and only 15% in patients with sporadic tumors. Among the 77 American patients with HNPCC investigated by Lynch *et al.* (1991) 20% were diagnosed as mucinous as compared with 8% in the control group. Jass *et al.* (1994) observed 19% of mucinous carcinomas among 140 HNPCCs *vs* 10% expected among sporadic colon cancers. A high percentage of mucinous carcinomas was observed in several small groups of HNPCC patients (Budd and Fink, 1981; Abusamra *et al.*, 1987; Purtilo *et al.*, 1987; Calmes *et al.*, 1992).

The recent findings on the genetic basis of HNPCC suggest that inherited mutations in ***hMSH2*** and ***hMLH1*** genes account for the majority of the HNPCC cases (Leach *et al.*, 1993; Parsons *et al.*, 1993; Aaltonen and Peltomäki, 1994; Loeb, 1994; Peltomäki, 1994; Smith, 1994). These genes encode the human homologues of bacterial proteins MutS and MutL, which are part of the base mismatch repair system. Loss of their function may explain the erroneous replication of microsatellites in hereditary colon cancers (Umar *et al.*, 1994). Since the frequency of replication error-positive (RER+) colon cancers is higher than that of HNPCCs, the defective mismatch repair may contribute to the development of both hereditary and sporadic colonic tumours (Kim *et al.*, 1994). The mutations at codons 12 or 13 in Ki-*ras* gene were found in 61% of HNPCC patients, a percentage higher than found in the group of sporadic cases (40%) (Aaltonen *et al.*, 1993). These morphological and genetic observations support the notion that a considerable percentage of the HNPCCs exhibit mucinous phenotype characterised by a high frequency of Ki-*ras* mutations.

### *Association with Crohn's disease and ulcerative processes*

Patients with Crohn's disease have several-fold increased risk of colorectal carcinoma as compared with the general population. In two studies 50% (Hamilton, 1985) or 29% (Choi and Zelig, 1994) of these carcinomas were identified as mucinous or mucinous and signet ring cell carcinomas respectively. Other groups did not report more frequent occurrence of mucinous tumours in patients with Crohn's disease (Lightdale *et al.*, 1975; Gyde *et al.*, 1980; Ekbom *et al.*, 1990). Conversely, the analysis of 120 mucinous carcinomas showed that only 15 tumours were associated with colitis or ulcerative colitis (Symonds and Vickery, 1976). These data indicate that the inflammatory process is not necessary but it may facilitate the preferential development of the mucinous phenotype.

### *High incidence after ionising irradiation*

Patients treated by radiotherapy of cancers localised in the abdomen (usually a total local dose in the range of 60 Gy fractionated over about 30 weeks), have an increased risk of developing colonic cancers. Mucinous carcinomas represent 26–58% of these tumours (Castro *et al.*, 1973; Kato *et al.*, 1981; Jao *et al.*, 1987). The development of mucinous carcinomas was also observed in 47% of rats after local irradiation of the colon with a single dose of 45 Gy (Denman *et al.*, 1978). Among the atomic bomb survivors who suffered a total dose below 1 Gy, the risk of colon cancer was increased but there was no frequency increase of mucinous phenotype (Nakatsuka *et al.*, 1992). Thus it is possible that radiation enteritis rather than radiation mutagenesis is the factor facilitating the development of the mucinous phenotype.

### Genetic alterations

### *Low frequency of p53 mutation and LOH*

The nuclear phosphoprotein p53 appears to function as a 'guardian of the genome' (Lane, 1992; Levine, 1993), arresting cells in $G_1$ phase in response to DNA damage and in some cases triggering cell death by apoptosis (Lane, 1993; Lee *et al.*, 1994). Further, recent data indicate that p53 may directly and indirectly stimulate DNA repair (Smith *et al.*, 1994). Mutation of the p53 gene is one of the most frequent genetic lesions associated with cancer (Greenblatt *et al.*, 1994). Mutations occur most commonly in four regions of the p53 gene ('hotspots'), highly conserved among species. The p53 gene mutation is frequently but not systematically (with concordancy of about 70%; Cripps *et al.*, 1994) related to accumulation of p53 protein. While the physiological concentrations of the wild-type p53 protein are immunohistochemically undetectable, the overexpression of the p53 molecule can usually be visualised. It is rare in adenomas but occurs in 50–73% of sporadic colon cancers (Van den Berg *et al.*, 1989; Purdie *et al.*, 1991; Scott *et al.*, 1991; Hanski *et al.*, 1992), indicating that it is a late event of colon carcinogenesis. The overexpression of p53 was detected with the monoclonal antibody PAb 1801 in 73% of non-mucinous colorectal adenocarcinomas but only in 25% of the mucinous carcinomas (Campo *et al.*, 1991). Similar results have been obtained with the polyclonal antibody CM-1 (72% and 36% respectively) (Hanski *et al.*, 1992), indicating that the difference was not due to a lost epitope. The frequency of mutations in non-selected colonic carcinomas detected by PCR-based techniques is 50–63% (Greenblatt *et al.*, 1994; Hamelin *et al.*, 1994*a*; Costa *et al.*, 1995). By contrast, the sequencing of relevant exons of p53 gene in the DNA isolated and amplified from mucinous carcinomas of the colon revealed mutations in only 25–31% of cases (Costa *et al.*, 1995; C Hanski *et al.*, unpublished), thus indicating that not only the overexpression but also the mutation of the p53 gene occurs less frequently in mucinous than in the non-mucinous tumours. The predominant type of mutation was GC→AT transitions, the most frequent type of p53 mutations in sporadic carcinomas. These results suggest that the alterations of the p53 gene in mucinous colorectal carcinomas are qualitatively similar, although less frequent than in non-mucinous cancer. These lesions seem therefore to be not essential for the development of the majority of mucinous colorectal tumours.

The inactivation of p53 function can occur not only through somatic mutation of the p53 gene but also by complex formation with viral oncogene products, cellular proteins or by alteration in subcellular localisation (Chang *et al.*, 1993; Zambetti and Levine, 1993). The normal protein binds to SV40 large T antigen, to the adenovirus protein E1B, the papilloma virus protein E6, as well as to the cellular protein MDM2 (Chang *et al.*, 1993). Binding of p53 to SV40 large antigen or E1B protein leads to an increased half-life of p53 while E6 proteins facilitate the degradation of p53 (Scheffner *et al.*, 1990; Werness *et al.*, 1990). Tumours resulting from this pathway may contain only wild-type p53 allele. Indeed, in both cervical carcinomas associated with papilloma virus and in sarcomas with MDM2 amplification, p53 mutations appear to be rare, whereas they are common in anogenital

Table I Incidence of mucinous colon carcinoma (Muc-CA) in different countries

| Colon carcinoma incidence per $10^5$ persons | Prevalence of Muc-CA (%) | Muc-CA incidence per $10^6$ persons | | No. of colon CA cases evaluated | Reference |
|---|---|---|---|---|---|
| 29.2 | 6.4 | 19 | Japan | 540 | Okuno *et al.* (1988)[a] |
| 29.2 | 6.6 | 19 | Japan | 662 | Yamamoto *et al.* (1993) (>50%) |
| 53 | 9 | 48 | Nova Scotia | 417 | Dajani *et al.* (1980) (>50%) |
| 56.3 | 11 | 62 | England | 669 | Umpleby *et al.* (1985) (>60%) |
| 48.5 | 10 | 49 | Norway | 534 | Halvorsen and Seim, (1988)[a] |
| 60.7 | 14 | 85 | Australia | 519 | Pihl *et al.* (1980) (>50%) |
| 33 | 15 | 50 | Finland | 75 | Mecklin *et al.* (1986) (>60%) |
| 79.2 | 15 | 119 | USA | 893 | Symonds and Vickery (1976) (>60%) |
| 14.6 | 19 | 28 | India | 118 | Suma and Nirmala (1992) (>50%) |
| 13 | 22 | 29 | Jordan | 141 | Dajani *et al.* (1980) (>50%) |

Incidence of all cancers is an estimate derived from the IARC statistics on cancer incidence in different countries (Waterhouse *et al.*, 1982). The incidence of mucinous carcinomas is calculated from the prevalence data in individual reports. The percentage of mucin in the sections used by each author to define the mucinous phenotype is given in brackets. [a]In tumours defined as mucinous the mucinous component was predominant.

malignancies not associated with virus and in sarcomas without MDM2 amplification (Chang *et al.*, 1993).

The mechanism underlying the formation of mucinous carcinoma in the presence of intact p53 is not known. The data obtained on human colorectal carcinomas are supported by observations made *in vitro*. Progression of the adenoma-derived cell line PC/AA to the mucinous malignant phenotype did not involve p53 protein overexpression, while progression to the adenocarcinoma phenotype was associated with the increase of cellular p53 protein expression (Williams *et al.*, 1993). Similarly, a spontaneous progression of a colonic adenoma cell line VACO-235 to mucinous carcinoma occurred without mutations in the p53 gene (Markowitz *et al.*, 1994).

It is of particular interest that, similarly to colorectal tumours, the mucinous carcinomas of the pancreas (Hoshi *et al.*, 1994; Zhang *et al.*, 1994), breast (Domagala *et al.*, 1993; Marchetti *et al.*, 1993) and ovary (Milner *et al.*, 1993; Rennison *et al.*, 1994) show either no alterations in the p53 gene nor in its expression, or the alterations are significantly less frequent than in the non-mucinous tumours of the same organs.

While the p53 gene, which is located on chromosome 17p, appears to be less frequently mutated in mucinous tumours, the less frequent loss of heterozygosity in mucinous than in non-mucinous tumours was observed not only on chromosome 17p (44% *vs* 88%) but also on chromosome 18q (47% *vs* 85%) (Kern *et al.*, 1989). Since the loss of heterozygosity on chromosome 17p or 18q, however, is generally less frequent in proximal than in the distal colon (Thibodeau *et al.*, 1993), the correlation of this property with the mucinous phenotype must be verified on selected tumours of either type from the proximal colon.

In tumours with non-mutated p53 the DNA index (which is the ratio of DNA content of malignant cells to that of normal cells) was reported to be lower than in those with mutated p53 (Hamelin *et al.*, 1994*a*), which would imply that mucinous tumours may have a lower DNA index than non-mucinous ones. Indeed, in mucinous tumours a higher incidence of diploid pattern (Kanagawa *et al.*, 1992) and a lower DNA index than in the non-mucinous tumours (Lanza *et al.*, 1994) were observed, the latter apparently being independent from tumour location (Lanza *et al.*, 1994).

### *High frequency of mutations in Ki-*ras *proto-oncogene*

Ki-*ras* protein p21 belongs to the family of GTP/GDP binding proteins with GTPase activity, which participate in transduction of mitogenic signals from the membrane to the cell nucleus (Lowy and Willumsen, 1993). Mutated ras proteins have a reduced GTPase activity and/or an increased dissociation rate of ras–GDP, leading to a prolonged mitogenic signal (Egan and Weinberg, 1993).

Single-point mutations in the Ki-*ras* proto-oncogene leading to substitution of critical amino acid residues in the p21 protein are sufficient to confer transforming properties to this gene (Reddy *et al.*, 1982). In human colorectal carcinogenesis the alterations of the Ki-*ras* gene appear to occur during the early steps of tumour formation, particularly during the development of adenomatous polyps (Farr *et al.*, 1988; Vogelstein *et al.*, 1988). The prevalence of Ki-*ras* mutations increases in adenomas at a more advanced stage of progression (Forrester *et al.*, 1987; Fearon and Vogelstein, 1990) and reaches 50% in non-selected carcinomas (Fearon and Vogelstein, 1990). In mucinous adenocarcinomas the codons 12 and 13 are affected in 65% of cases, while in non-mucinous ones the mutations occur in only 33% of these loci (Laurent Puig *et al.*, 1991). Of interest, in mucinous ovarian tumours the prevalence of Ki-*ras* mutations is higher than in the non-mucinous ones (Enomoto *et al.*, 1991; Ichikawa *et al.*, 1994), indicating that also in ovarian tumours the high frequency of Ki-*ras* mutation is preferentially associated with the mucinous phenotype.

### *Amplification of c-*myc *proto-oncogene*

The c-myc-coded dimeric nuclear phosphoprotein binds to DNA and regulates gene transcription; therefore it has potential importance as a determinant of the proliferation state of the cell (Kretzner *et al.*, 1985; Dang, 1991; Marcu *et al.*, 1992). It has been proposed that abnormal myc expression would alter the regulation of cellular genes, rendering cells more susceptible to malignant transformation. The dominant action of another oncogene or the loss of a tumour-suppressor gene would then accelerate or promote tumorigenesis (Hunter, 1991). The *myc* gene co-operates with *ras* to transform rat fibroblasts, rat embryo cells and human epithelial cells (Marcu *et al.*, 1992), but deregulated *myc* expression alone is not sufficient to elicit malignant phenotype in the absence of secondary events. Organ culture experiments have shown further that activated *ras* and *myc* genes together can induce malignant tumours without p53 mutation (Lu *et al.*, 1992). The activation of the c-*myc* gene may be of relevance for progression of colonic tumours since the increase in expression of c-*myc* mRNA and its protein product correlates with dysplasia grade in adenomas and with the progression from adenoma to colon carcinoma (Erisman *et al.*, 1985; Rothberg *et al.*, 1985; Sikora *et al.*, 1987; Finley *et al.*, 1989; Agnantis *et al.*, 1991; Pavelic *et al.*, 1992; Tulchin *et al.*, 1992; Hanski *et al.*, 1994; Sato *et al.*, 1994).

The c-*myc* proto-oncogene is present as a single copy gene in the normal human genome. In 54% of mucinous colorectal carcinomas in a group of 13 American patients a modest amplification of the c-*myc* gene was found, as compared with 7% (2/29) in moderately to well-differentiated non-mucinous carcinomas (Heerdt *et al.*, 1991). These authors associated c-*myc* amplification with the more aggressive, malignant phenotype (Heerdt *et al.*, 1991), a finding corroborated by other workers (Kozma *et al.*, 1994). In a study of 100 Asian patients with colorectal cancer, however, no c-*myc* gene amplification was detected (Smith *et al.*, 1993). Further, the tumours located distal to the transverse colon (the majority of which are non-mucinous) overexpress c-*myc* more frequently than the proximal tumours (Rothberg, 1987). The slight increase in gene copy number detected in the American patients (Heerdt *et al.*, 1991) may have little effect on the c-*myc* message expression, however, it indicates a different frequency of proto-oncogene lesion in mucinous and non-mucinous tumours in this group of patients.

### *Frequent overexpression of mucin MUC2*

MUC2 is a well-characterised intestinal mucin, present predominantly in the small intestine and in the colon (Ho *et al.*, 1993). Strong expression is observed in 72% of the mucinous but only in 21% of non-mucinous colonic carcinomas. Also 40–48% of colonic adenomas show strong MUC2 expression. The comparison of expression in the premalignant and malignant colonic tissues of the same specimens indicated that MUC2 overexpression occurring in the adenoma tissue is maintained or increased if the adenoma progresses to mucinous carcinoma. If, however, the adenoma develops into a non-mucinous adenocarcinoma, the expression frequently decreases below the normal level (Blank *et al.*, 1994). Thus the overexpression of MUC2 is occurring already in the premalignant stage of the adenoma–carcinoma sequence and remains a characteristic property of the mucinous phenotype of colorectal tumours (Ho *et al.*, 1993; Blank *et al.*, 1994).

The mechanisms responsible for MUC2 overexpression in mucinous carcinomas is not known. The overexpression of MUC2 in colon adenocarcinoma cells *in vitro* can be induced by 12-*O*-tetradecanoyl phorbol acetate (TPA) or forskolin. Both inducers have been shown to operate by triggering their respective signal transduction pathways, via protein kinase C-(TPA) or protein kinase A-(forskolin) (Velcich and Augenlicht, 1993). Whether these transduction pathways are involved in the MUC2 overexpression *in vivo*, has not been investigated.

### *More frequent loss or low expression of major histocompatibility complex (MHC) class I molecules*

The products of the MHC play an important role in the regulation of several immune functions: MHC class I molecules serve as restriction elements for T-cell-mediated cytotoxicity, whereas MHC class II molecules are required for the presentation of antigens to autologous helper T cells, MHC class I molecules are strongly expressed on morphologically normal colonic epithelial cells and in colonic adenomas (Van den Ingh *et al.*, 1987; Garrido *et al.*, 1993). The investigation of 152 patients indicated that about 44% of non-selected carcinomas exhibit a reduction or loss of MHC class I molecules (Möller *et al.*, 1991). The same study indicated that the low expression or loss of MHC class I antigens is more frequent in mucinous than in non-mucinous tumours, a finding corroborating previous data from a smaller group of patients (Van den Ingh *et al.*, 1987).

The mechanism of MHC class I loss in carcinomas is not known. Only two cumulative mutations in $\beta_2$-microglobulin ($\beta_2$-M) genes would be sufficient to induce complete loss of MHC class I antigens. MHC class I-negative colon carcinomas lack also $\beta_2$-M expression which was interpreted as an indication that this may be the mechanism of MHC class I loss in these tumours (Momburg and Koch, 1989; Cabrera *et al.*, 1991).

### *Less frequent loss of MHC class II molecules*

Most normal epithelia, including colon, are MHC class II negative (McDonald and Jewell, 1987). In colonic tissue the majority of premalignant lesions acquire *de novo* MHC class II expression, and severely dysplastic colonic adenomas are positive in 100% of cases. The progression to non-mucinous adenocarcinoma is associated with a loss of expression of MHC class II molecules in 68% of cases while in mucinous carcinomas this loss is observed in only 37%. Thus the mucinous carcinomas express MHC class II molecules about twice as frequently as the non-mucinous ones (Garrido *et al.*, 1993).

### *Microsatellite instability*

A subset of sporadic colorectal cancers and most of the hereditary non-polyposis colorectal cancers (HNPCC) exhibit widespread alterations of short, repeated sequences (microsatellites) distributed throughout the genome (Ionov *et al.*, 1993). The alteration of microsatellite length (or sequence) that occurs during colon carcinogenesis is associated with mutations in mismatch repair genes hMSH2, hMLH1, hPMS1 and hPMS2, which yield defective repair proteins unable to correct replication errors (Bronner *et al.*, 1994; Loeb, 1994; Papadopoulos *et al.*, 1994; Peltomäki, 1994; Eshleman and Markowitz, 1995). Sporadic colorectal cancers show replication errors in di- tri- or tetranucleotide loci in 13–16.5% of tumours (Aaltonen *et al.*, 1993; Lothe *et al.*, 1993; Hamelin *et al.*, 1994*b*; Kim *et al.*, 1994). Among the parameters that correlate with microsatellite instability in these tumours is the proximal location, extracellular mucin production and a trend towards less frequent p53 gene product overexpression, as detected by immunohistochemistry (Hamelin *et al.*, 1994*b*; Kim *et al.*, 1994). While among non-mucinous tumours the frequency of replication error-positive (RER+) phenotype was 9% (12/128), 66% of mucinous tumors (6/9) were RER+ (Kim *et al.*, 1994). There is no relationship between p53 point mutations and microsatellite instability (Hamelin *et al.*, 1994*b*). The question whether the lesions in mismatch repair system substitute the p53 mutations and represent an independent carcinogenesis pathway associated with the mucinous phenotype warrants further investigation. Further, the analysis of a large number of tumours from both the distal and proximal colon is necessary to establish how far the relative preponderance of mucinous tumours in the proximal colon contributes to the observed correlation.

### Conclusions and aetiological implications

The recent genetic evidence in combination with the previous data pose the question if the mucinous colorectal carcinoma is a distinct genetic entity, different from the non-mucinous carcinoma. Both types of colonic cancer differ not only in their morphology but also in their localisation, incidence and the pattern of genetic lesions. An intriguing observation is that mucinous carcinomas not only of the colon but also of other organs (breast, pancreas, ovary) appear to share certain genetic properties. In these tumours the frequency of p53 mutations is lower and the frequency of Ki-*ras* mutations is higher than in the corresponding non-mucinous tumours of the same organs, suggesting a 'mucinous phenotype-related' pathway of carcinogenesis. Since the definition of mucinous tumours is based on quantitative rather than qualitative criterion, this remains a hypothesis until the lesion(s) common to all mucinous carcinomas, responsible for the overexpression of mucin genes and possibly related to the 'mucinous pathway of carcinogenesis' are identified.

Different lesions may be due to a distinct aetiology of the mucinous tumours. The role of the aberrantly expressed mucin genes in this process is not clear since different mucins, coded by genes localised on separate chromosomes, predominate in different organs. One parameter emerging from experimental studies as well as from retrospective analysis of patient history is intestinal inflammation as a factor facilitating the preferential development of the mucinous phenotype in the colon. A comparative analysis of the genetic lesions of mucinous and non-mucinous tumours would further our understanding of factors affecting differentiation in colorectal cancer. Identification of the genetic changes characteristic of the mucinous phenotype may help not only to better understand its aetiology but possibly establish markers for detection and surveillance of the high-risk population.

**Acknowledgements**

This work was supported by the Deutsche Krebshilfe, Project No. W 17/92/Ri2 and the Deutsche Forschungsgemeinschaft, Project No. Ha 1520/5-2. The author is indebted to Dr JR Jass, Department of Pathology, University of Auckland, New Zealand and Dr R Riddell, Department of Pathology, McMaster University, Canada for reading the manuscript and valuable comments.

### References

AALTONEN LA AND PELTOMÄKI P. (1994). Genes involved in hereditary nonpolyposis colorectal carcinoma. *Anticancer Res.*, **14,** 1657–1660.

AALTONEN LA, PELTOMÄKI P, LEACH FS, SISTONEN P, PYLKKÄNEN L, MECKLIN JP, JÄRVINEN H, POWELL SM, JEN J, HAMILTON SR, PETERSEN GM, KINZLER KW, VOGELSTEIN B AND DE LA CHAPELLE A. (1993). Clues to the pathogenesis of familial colorectal cancer. *Science*, **260,** 812–816.

ABUSAMRA H, MAXIMOVA S, BAR-MEIR S, KRISPIN M AND ROTMENSCH HH. (1987). Cancer family syndrome of Lynch. *Am. J. Med.*, **83,** 981–983.

AGNANTIS NJ, APOSTOLIKAS N, SFICAS C, ZOLOTA V AND SPANDIDOS DA. (1991). Immunohistochemical detection of ras p21 and c-myc p62 in colonic adenomas and carcinomas. *Hepato-Gastroenterol.*, **38,** 239–242.

ANGEL C, PRATT C, RAO B, SCHELL M, PARHAM D, LOBE T AND FLEMING I. (1992). Carcinoembryonic antigen and carbohydrate 19-9 antigen as markers for colorectal carcinoma in children and adolescents. *Cancer*, **69,** 1487–1491.

BLANK M, KLUSSMAN E, KRÜGER-KRASAGAKES S, SCHMITT-GRÄFF A, STOLTE M, BORNHOEFT G, STEIN H, XING PX, MCKENZIE IFC, VERSTIJNEN CPHJ, RIECKEN EO AND HANSKI C. (1994). Expression of MUC2-mucin in colorectal adenomas and carcinomas of different histological types. *Int. J. Cancer*, **59,** 301–306.

BRONNER CE, BAKER SM, MORRISON PT, WARREN G, SMITH LG, LESCOE MK, KANE M, EARABINO C, LIPFORD J, LINDBLOM A, TANNERGÄRD P, BOLLAG RJ, GODWIN AR, WARD DC, NORDENSKJOLD M, FISHEL R, KOLODNER R AND LISKAY M. (1994). Mutation in the DNA mismatch repair gene homologue hMLH1 is associated with hereditary non-polyposis colon cancer. *Nature*, **368,** 258–259.

BUDD DC AND FINK DL. (1981). Mucoid colonic carcinoma as an autosomal-dominant inherited syndrome. *Arch. Surg.*, **116,** 901–905.

CABRERA T, CONCHA A, RUIZ-CABELLO F AND GARRIDO F. (1991). Loss of HLA heavy chain and $\beta_2$-microglobulin in HLA negative tumours. *Scand. J. Immunol.*, **34,** 147–152.

CALMES JM, RUTZ HP, SUARDET L AND GIVEL JC. (1992). Hereditary colorectal cancer: observations of a family study. *Helvet. Chirurg. Acta*, **59,** 349–354.

CAMPO E, DE LA CALLE-MARTIN O, MIGUEL R, PALACIN A, ROMERO M, FABREGAT V, VIVES J, CARDESA A AND YAGUE J. (1991). Loss of heterozygosity of p53 gene and p53 proteinexpression in human colorectal carcinomas. *Cancer Res.*, **51,** 4436–4442.

CASTRO E, ROSEN P AND QUAN S. (1973). Carcinoma of large intestine in patients irradiated for carcinoma of cervix and uterus. *Cancer*, **31,** 45–52.

CHANG F, SYRJÄNEN S, TERVAHAUTA A AND SYRJÄNEN K. (1993). Tumorigenesis associated with the p53 tumour suppressor gene. *Br. J. Cancer*, **68,** 653–661.

CHOI PM AND ZELIG MP. (1994). Similarities of colorectal cancer in Crohn's disease and ulcerative colitis: implications for carcinogenesis and prevention. *Gut*, **35,** 950–954.

COSTA A, MARASCA R, VALENTININS B, SAVARINO M, FARANDA A, SILVESTRINI R AND TORELLI G. (1995). p53 gene point mutations in relation to p53 nuclear protein accumulation in colorectal cancers. *J. Pathol.*, **176,** 45–53.

CRIPPS KJ, PURDIE CA, CARDER PJ, WHITE S, KOMINE K, BIRD CC AND WYLLIE A-H. (1994). A study of stabilisation of p53 protein versus point mutation in colorectal carcinoma. *Oncogene*, **9,** 27739–27743.

DAJANI YF, ZAYID I, MALATJALIAN DA AND KAMAL MF. (1980). Colorectal cancer in Jordan and Nova Scotia. A comparative epidemiologic and histopathologic study. *Cancer*, **46,** 420–426.

DANG CV. (1991). c-myc oncoprotein function. *Biochim. Biophys. Acta*, **1072,** 103–113.

DENMAN DL, KIRCHNER FR AND OSBORNE JW. (1978). Induction of colonic adenocarcinoma in the rat by X-irradiation. *Cancer Res.*, **38,** 1899–1905.

DOMAGALA W, HAREZGA B, SZADOWSKA A, MARKIEWSKI M, WEBER K AND OSBORN M. (1993). Nuclear p53 protein accumulates preferentially in medullary and high-grade ductal but rarely in lobular breast carcinomas. *Am. J. Pathol.*, **142,** 669–674.

EGAN SE AND WEINBERG RA. (1993). The pathway to signal achievement. *Nature*, **365,** 781–782.

EKBOM A, HELMICK C, ZACK M AND ADAMI HO. (1990). Increased risk of large-bowel cancer in Crohn's disease with colonic involvement. *Lancet*, **336,** 357–359.

ENOMOTO T, WEGHORST CM, INOUE M, TANIZAWA O AND RICE JM. (1991). K-ras activation occurs frequently in mucinous adenocarcinomas and rarely in other common epithelial tumors of the human ovary. *Am. J. Pathol.*, **139,** 777–785.

ERISMAN MD, ROTHBERG PG, DIEHL RE, MORSE CC, SPANDORFER JM AND ASTRIN SM. (1985). Deregulation of c-*myc* gene expression in human colon carcinoma is not accompanied by amplification or rearrangement of the gene. *Mol. Cell Biol.*, **5,** 1969–1976.

ESHLEMAN JR AND MARKOWITZ SD. (1995). Microsatellite instability in inherited and sporadic neoplasms. *Curr. Opinion Oncol.*, **7,** 83–89.

FARR CJ, MARSHALL CJ, EASTY DJ, WRIGHT NA, POWELL SC AND PARASKEVA C. (1988). A study of *ras* gene mutations in colonic adenomas from familial polyposis coli patients. *Oncogene*, **3,** 673–678.

FEARON ER AND VOGELSTEIN B. (1990). A genetic model for colorectal carcinogenesis. *Cell*, **61,** 759–767.

FERGUSON EJ AND OBI L. (1971). Carcinoma of the colon and rectum in patients up to 25 years of age. *Am. Surg.*, **37,** 181–189.

FINLEY GG, SCHULZ NT, HILL SA, GEISER JR, PIPAS JM AND MEISLER AI. (1989). Expression of the *myc* gene family in different stages of human colorectal cancer. *Oncogene*, **4,** 963–971.

FORRESTER K, ALMOGUERA C, HAN K, GRIZZLE WE AND PERUCHO M. (1987). Detection of high incidence of K-*ras* oncogenes during human colon tumorigenesis. *Nature*, **327,** 298–303.

GARRIDO F, CABRERA T, CONCHA A, GLEW S, RUIZ-CABELLO F AND STERN PL. (1993). Natural history of HLA expression during tumor development. *Immunol. Today*, **14,** 491–499.

GREENBLATT MS, BENNETT WP, HOLLSTEIN M AND HARRIS CC. (1994). Mutations in the p53 tumor suppressor gene: clues to cancer etiology and molecular pathogenesis. *Cancer Res.*, **54,** 4855–4878.

GYDE SN, PRIOR P, MACARTNEY JC, THOMPSON H, WATERHOUSE JAH AND ALLAN RN. (1980). Malignancy in Crohn's disease. *Gut*, **21,** 1024–1029.

HALVORSEN TB AND SEIM E. (1988). Influence of mucinous components on survival in colorectal carcinomas: a multivariate analysis. *J. Clin. Pathol.*, **41,** 1068–1072.

HAMELIN R, LAURENT-PUIG P, OLSCHWANG S, JEGO N, ASSELAIN B, REMVIKOS T, GIRODET J, SALMON RJ AND THOMAS G. (1994*a*). Association of p53 mutations with short survival in colorectal cancer. *Gastroenterology*, **106,** 42–48.

HAMELIN R, LAURENT-PUIG P, OLSCHWANG S, SALMON RJ AND THOMAS G. (1994*b*). Genetic instability of microsatellites in human colon cancer. *Gastroenterology*, **106** (4 suppl), A390.

HAMILTON SR. (1985). Colorectal carcinoma in patients with Crohn's disease. *Gastroenterology*, **89,** 398–407.

HANSKI C, BORNHOEFT G, SHIMODA T, HANSKI ML, LANE D, STEIN H AND RIECKEN EO. (1992). Expression of p53 protein in invasive colorectal carcinomas of different histological type. *Cancer*, **70,** 2772–2777.

HANSKI C, ODEFEY U, OGOREK D, WANG J, BORNHOEFT G AND RIECKEN EO. (1994). The overexpression of the sialyl-Lewis$^x$ moiety is an independent and a more consistent marker of colon carcinogenesis than the overexpression of c-*myc* and Ki-*ras* oncogenes. *Int. J. Oncol.*, **4,** 993–1000.

HANSKI C, BORNHOEFT G, SHIMODA T, HANSKI ML, LANE D, STEIN H AND RIECKEN EO. (1992). Expression of p53 protein in invasive colorectal carcinomas of different histological type. *Cancer*, **70**, 2772-2777.

HANSKI C, ODEFEY U, OGOREK D, WANG J, BORNHOEFT G AND RIECKEN EO. (1994). The overexpression of the sialyl-Lewis$^x$ moiety is an independent and a more consistent marker of colon carcinogenesis than the overexpression of c-*myc* and Ki-*ras* oncogenes. *Int. J. Oncol.*, **4**, 993-1000.

HANSKI C, BLANK M, HANSKI ML AND RIECKEN EO. (1995). Phenotypic and clinicopathological pecularities of mucinous colonic carcinoma. In *Malignomentstehung und chronische Entzündungen im Gastrintestinaltrakt-Neue Konzepte*. Falk Symposium No. 81. Riechen ED, Zeitz M, Stallmach A, Heise W (eds). pp 122-133. Kluwer Academic.

HEERDT BG, MOLINAS S, DEITCH D AND AUGENLICHT LH. (1991). Aggressive subtypes of human colorectal tumors frequently exhibit amplification of the c-*myc* gene. *Oncogene*, **6**, 125-129.

HERMANEK P, GUGGENMOOS-HOLZMANN I AND GALL FP. (1989). Prognostic factors in rectal carcinoma. A contribution to the further development of tumour classification. *Dis. Colon Rectum*, **32**, 593-599.

HO SB, NIEHANS GA, LYFTOGT C, YAN PS, CHERWITZ DL, GUM ET, DAHIYA R AND KIM YS. (1993). Heterogeneity of mucin gene expression in normal and neoplastic tissues. *Cancer Res.*, **53**, 641-651.

HOSHI T, IMAI M AND OGAWA K. (1994). Frequent K-*ras* mutations and absence of p53 mutations in mucin-produding tumors of the pancreas. *J. Surg. Oncol.*, **55**, 84-91.

HUNTER T. (1991). Cooperation between oncogenes. *Cell*, **64**, 249-270.

ICHIKAWA Y, NISHIDA M, SUZUKI H, YOSHIDA S, TSUNODA H, KUBO T, UCHIDA K AND MIWA M. (1994). Mutation of K-*ras* protooncogene is associated with histological subtypes in human mucinous ovarian tumors. *Cancer Res.*, **54**, 33-35.

IONOV Y, PEINADO MA, MALKHOSYAN S, SHIBATA D AND PERUCHO M. (1993). Ubiquitious somatic mutations in simple repeated sequences reveal a new mechanism for colonic carcinogenesis. *Nature*, **363**, 558-561.

JAO SW, BEAR RWJ, REIMAN HM, GUNDERSON LL AND ILSTRUP DM. (1987). Colon and anorectal cancer after pelvic irradiation. *Dis. Colon Rectum*, **30**, 953-958.

JASS JR. (1993). Evolution of hereditary bowel cancer. *Mutation Res.*, **290**, 13-25.

JASS JR AND SOBIN LH. (1990). *Histological Typing of Intestinal Tumors*. Springer Verlag: Berlin.

JASS JR, SMYRK TC, STEWART SM, LANE MR, LANSPA SJ AND LYNCH HT. (1994). Pathology of hereditary non-polyposis colorectal cancer. *Anticancer Res.*, **14**, 1631-1634.

KANAGAWA T, OKAJIMA K, MIZUTANI H, TOYODA M, MARUKAWA O, NISHINO H AND LEE K. (1992). Clinicopathological studies of mucinous carcinoma of the large bowel. *J. Jpn. Soc. Colo-Proct.*, **45**, 837-842.

KATO Y, YANAGISAWA A, YAMADA Y, YAGASAKI K, NAKAMURA K, SUGANO H AND TAKAHASHI T. (1981). A pathohistological study of cancer of the colon and changes in the mucosal epithelium after radiotherapy. (Meeting Abstract). Proceedings of the 15th Meeting of the Jpn Res. Soc. for Cancer of the Colon and Rectum, Kagoshima, Japan. 24 July 1981. p 66. Jpn. Res Soc. for Cancer of the Colon and Rectum: Osaka, Japan.

KERN SE, FEARON ER, TERSMETTE KWF, ENTERLINE JP, LEPPERT M, NAKAMURA Y, WHITE, R, VOGELSTAIN B AND HAMILTON DSR. (1989). Allelic loss in colorectal carcinoma. *JAMA*, **261**, 3099-3103.

KIM H, JEN J, VOGELSTEIN B AND HAMILTON SR. (1994). Clinical and pathological characteristics of sporadic carcinomas with DNA replication errors in microsatellite sequences. *Am. J. Pathol.*, **145**, 148-156.

KOH SJ, CACES JN AND JOHNSON WW. (1980). Colorectal carcinoma in children. *Lab. Invest.*, **42**, 174.

KOZMA L, KISS I, SZAKALL S AND EMBER I. (1994). Investigation of c-*myc* oncogene amplification in colorectal cancer. *Cancer Lett.*, **81**, 165-169.

KRETZNER L, BLACKWOOD EM AND EISENMAN RN. (1992). Myc and Max proteins possess distinct transcriptional activities. *Nature*, **359**, 426-429.

LANE DP. (1992). p53, guardian of the genome. *Nature*, **358**, 15-16.

LANE DP. (1993). Cancer. A death in the life of p53 (news comment). *Nature*, **362**, 786-787.

LANZA G, MAESTRI I, BALLOTA MR, DUBINI A, AND CAVAZZINI L. (1994). Relationship of nuclear DNA content to clinicopathological features in colorectal cancer. *Modern Pathol.*, **7**, 161-165.

LAURENT-PUIG P, OLSCHWANG S, DELATTRE O, VALIDIRE P, MELOT T, MOSSERI V, SALMON RJ AND THOMAS G. (1991). Association of Ki-*ras* mutation with differentiation and tumorformation pathways in colorectal carcinoma. *Int. J. Cancer*, **49**, 220-223.

LEACH FS, NICOLAIDES NC, PAPADOPOULOS N, LIU B, JEN J, PARSONS R, PELTOMÄKI P, SISTONEN P, AALTONEN LA, NYSTRÖM-LAHTI M, GUAN X-Y, ZHANG J, MELTZER PS, YU J-W, KAO F-T, CHEN DJ, CEROSALETTI KM, FOURNIER REK, TODD S, LEWIS T, LEACH RJ, NAYLOR SL, WEISSENBACH J, MECKLIN J-P, JÄRVINEN H, PETERSEN GM, HAMILTON SR, GREEN J, JASS J, WATSON P, LYNCH HT, TRENT JM, DE LA CHAPELLE A, KINZLER KW AND VOGELSTEIN B. (1993). Mutations of a mutS homolog in hereditary nonpolyposis colorectal cancer. *Cell*, **75**, 1215-1225.

LEE JM, ABRAHAMSON JLA AND BERNSTEIN A. (1994). DNA damage, oncogenesis and the p53 tumour-suppressor gene. *Mut. Res.*, **307**, 573-581.

LEVINE AJ. (1993). The tumor suppressor genes. *Annu. Rev. Biochem.*, **62**, 623-651.

LIGHTDALE CJ, STERNBERG SS, POSNER G AND SHERLOCK P. (1975). Carcinoma complicating Crohn's disease. Report of seven cases and review of the literature. *Am. J. Med.*, **59**, 262-268.

LOEB LA. (1994). Microsatellite instability: Marker of a mutator phenotype in cancer. *Cancer Res.*, **54**, 5059-5063.

LOTHE RA, PELTOMAKI P, MELING GI, AALTONEN LA, NYSTROM-LAHTI M, PYLKKANEN L, HEIMDAL K, ANDERSEN TI, MOLLER P, ROGNUM TO, FOSSA SD, HALDORSEN T, LANGMARK F, BROGGER A, DE LA CHAPELLE A AND BORRESEN A. (1993). Genomic instability in colorectal cancer: Relationship to clinicopathological variables and family history. *Cancer Res.*, **53**, 5849-5852.

LOWY DR AND WILLUMSEN BM. (1993). Function and regulation of *ras*. *Annu. Rev. Biochem.*, **62**, 851-891.

LU X, PARK SH, THOMPSON TC AND LANE DP. (1992). ras-induced hyperplasia occurs with mutation of p53, but activated *ras* and *myc* together can induce carcinoma without p53 mutation. *Cell*, **70**, 153-161.

LYNCH HT, LANSPA S, SMYRK T, BOMAN B, WATSON P AND LYNCH J. (1991). Hereditary nonpolyposis colorectal cancer (Lynch syndromes I & II). Genetics, pathology, natural history, and cancer control, part I. *Cancer Genet. Cytogenet.*, **53**, 143-160.

LYNCH HT, SMYRK TC, WATSON P, LANSPA SJ, LYNCH JF, LYNCH PM, CAVALIERI RJ AND BOLAND CR. (1993). Genetics, natural history, tumor spectrum, and pathology of hereditary nonpolyposis colorectal cancer: An updated review. *Gastroenterology*, **104**, 1535-1549.

LYNCH TH AND LYNCH JF. (1994). 25 years of HNPCC. *Anticancer Res.*, **14**, 11617-11624.

MCDONALD GB AND JEWELL DP. (1987). Class II antigen (HLA-DR) expression by intestinal epithelial cells in inflammatory diseases of colon. *J. Clin. Pathol.*, **40**, 312-317.

MARCHETTI A, BUTTITTA F, PELLEGRINI S, CAMPANI D, DIELLA F, CECCHETTI D, CALLAHAN R AND BISTOCCHI M. (1993). p53 mutations and histological type of invasive breast carcinoma. *Cancer Res.*, **53**, 4665-4669.

MARKOWITZ S, MYEROFF L, COOPER M, TRAICOFF J, KOCHERA M, LUTTERBAUGH J, SWIRIDUK M AND WILSON J. (1994). A benign cultured colon adenoma bears three genetically altered colon cancer oncogenes, but progresses to tumorigenicity and transforming growth factor-beta independence without inactivating the p53 tumor suppressor gene. *J. Clin. Invest.*, **93**, 1005-1013.

MARCU KB, BOSSONE SA AND PATEL AJ. (1992). *myc* function and regulation. *Annu. Rev. Biochem.*, **61**, 809-860.

MECKLIN JP, SIPPONEN P AND JARVINEN HJ. (1986). Histopathology of colorectal carcinomas and adenomas in cancer family syndrome. *Dis. Colon Rectum*, **29**, 849-853.

MILNE D. (1994). Right or left, right or wrong? Debate whirls over colorectal cancer distribution. *J. Natl Cancer Inst.*, **86**, 1442-1443.

MILNER BJ, ALLAN LA, ECCLES DM, KITCHENER HC, LEONARD RC, KELLY KF, PARKIN DE AND HAITES NE. (1993). p53 mutation is a common genetic event in ovarian carcinoma. *Cancer Res.*, **53**, 2128-2132.

MOMBURG F AND KOCH S. (1989). Selective loss of $\beta_2$-microglobulin mRNA in human colon carcinoma. *J. Exp. Med.*, **169**, 309–314.

MÖLLER P, MOMBURG F, KORETZ K, MOLDENHAUER G, HERFARTH C, OTTO HF, HÄMMERLING GJ AND SCHLAG P. (1991). Influence of major histocompatibility complex class I and II antigens on survival in colorectal carcinoma. *Cancer Res.*, **51**, 729–736.

NAKATSUKA H, SHIMIZU Y, YAMAMOTO T, SEKINE I, EZAKI H, TAHARA E, TAKAHASHI M, SHIMOYAMA T, MOCHINAGA N, TOMITA M, TSUCHIYA R AND LAND C. (1992). Colorectal cancer incidence among atomic bomb survivors, 1950–80. *J. Radiat. Res.*, **33**, 342–361.

ODONE V, CHANG L, CACES J, GEORGE S AND PRATT C. (1982). The natural history of colorectal carcinoma in adolescents. *Cancer*, **49**, 1716–1720.

OKUNO M, IKEHARA T, NAGAYAMA M, KATO Y, YUI S AND UMEYAMA K. (1988). Mucinous colorectal carcinoma: clinical pathology and prognosis. *Am. Surg.*, **54**, 681–685.

PAPADOPOULOS N, NICOLAIDES NC, WEI YF, RUBEN SM, CARTER KC, ROSEN CA, HASELTINE WA, FLEISCHMANN RD, FRASER CM, ADAMS MD, VENTER JC, HAMILTON SR, PETERSEN GM, WATSON P, LYNCH HT, PELTOMÄKI P, MECKLIN J-P, DE LA CHAPELLE A, KINZLER KW AND VOGELSTEIN B. (1994). Mutation of a mutL homolog in hereditary colon cancer. *Science*, **263**, 1625–1629.

PARSONS R, LI GM, LONGLEY MJ, FANG W, PAPADOPOULOS N, JEN J, DE LA CHAPELLE A, KINZLER KW, VOGELSTEIN B AND MODRICH P. (1993). Hypermutability and mismatch repair deficiency in RER$^+$ tumor cells. *Cell*, **75**, 1227–1236.

PAVELIC ZP, PAVELIC L, KUVELKAR R AND GAPANY SR. (1992). High c-myc protein expression in benign colorectal lesions correlates with the degree of dysplasia. *Anticancer Res.*, **12**, 171–176.

PELTOMÄKI PT. (1994). Genetic basis of hereditary nonpolyposis colorectal carcinoma (HNPCC). *Ann. Med.*, **26**, 215–219.

PIHL E, NAIRN RC, HUGHES ESR, CUTHBERTSON AM AND ROLLO AJ. (1980). Mucinous colorectal carcinoma: Immunopathology and prognosis. *Pathology*, **12**, 439–447.

PRATT C, PARHAM D, RAO B AND FLEMING I. (1987). Adolescent colon carcinoma, colonic polyps and neurofibromatosis. *Proc. Annu. Meet. Am. Assoc. Cancer Res.*, **28**, 254.

PURDIE C, O'GRADY J, PIRIS J, WYLLIE A AND BIRD C. (1991). p53 expression in colorectal tumors. *Am. J. Pathol.*, **138**, 807–813.

PURTILO DT, GEELHOED GW, LI FP, YANG JP, THURBER WA, DARRAH J AND CASSEL C. (1987). Mucinous colon carcinoma in a black family. *Cancer Genet. Cytogenet.*, **24**, 11–15.

REDDY EP, REYNOLDS RK, SANTOS E AND BARBACID M. (1982). A point mutation is responsible for the acquisition of transforming properties by the T24 human bladder carcinoma oncogene. *Nature*, **300**, 149–152.

RENNINSON J, BAKER BW, MCGOWN AT, MURPHY D, NORTON JD, FOX BW AND CROWTHER D. (1994). Immunohistochemical detection of mutant p53 protein in epithelial ovarian cancer using polyclonal antibody CM1: correlation with histopathology and clinical features. *Br. J. Cancer*, **69**, 609–612.

ROTHBERG PG. (1987). The role of the oncogene *c-myc* in sporadic large bowel cancer and familial polyposis coli. *Semin. Surg. Oncol.*, **3**, 152–158.

ROTHBERG PG, SPANDORFER JM, ERISMAN MD, STAROSCIK RN, SEARS HF, PETERSON RO AND ASTRIN SM. (1985). Evidence that c-myc expression defines two genetically distinct forms of colorectal adenocarcinoma. *Br. J. Cancer*, **52**, 629–632.

SASAKI O, ATKIN WS AND JASS JR. (1987). Mucinous carcinoma of the rectum. *Histopathology*, **11**, 259–272.

SATO K, MIYAHARA M, SAITO T AND KOBAYASHI M. (1994). C-myc mRNA overexpression is associated with lymph node metastasis in colorectal cancer. *Eur. J. Cancer*, **30**, 1113–1117.

SCHEFFNER M, WERNESS BA, HUIBREGTSE JM, LEVINE AJ AND HOWLEY PM. (1990). The E6 oncoprotein encoded by human papillomavirus types 16 and 18 promotes the degeneration of p53. *Cell*, **63**, 1129–1136.

SCOTT N, SAGAR P, STEWART J, BLAIR G, DIXON M AND QUIRKE P. (1991). p53 in colorectal cancer: clinicopathological correlation and prognostic significance. *Br. J. Cancer*, **63**, 317–319.

SIKORA K, CHAN S, EVAN G, GABRA H, MARKHAM N, STEWART J AND WATSON J. (1987). *c-myc* oncogene expression in colorectal cancer. *Cancer*, **59**, 1289–1295.

SMITH DR, MYINT T AND GOH HS. (1993). Over-expression of the *c-myc* proto-oncogene in colorectal carcinoma. *Br. J. Cancer*, **68**, 407–413.

SMITH ML, CHEN IT, ZHAN Q, BAE I, CHEN CY, GILMER TM, KASTAN MB, O'CONNOR PM AND FORNACE JR, AJ. (1994). Interaction of the p53-regulated protein Gadd 45 with proliferating cell nuclear antigen. *Science*, **266**, 1376–1380.

SMITH RG. (1994). Hereditary predisposition to colorectal cancer: New insights. *Am. J. Med. Sci.*, **308**, 295–308.

SUMA KS AND NIRMALA V. (1992). Mucinous component in colorectal carcinoma-prognostic significance: a study in a south Indian population. *J. Surg. Oncol.*, **51**, 60–64.

SUNBLAD AS AND PAZ RA. (1982). Mucinous carcinomas of the colon and rectum and their relation to polyps. *Cancer*, **50**, 2504–2509.

SYMONDS DA AND VICKERY JAL. (1976). Mucinous carcinoma of the colon and rectum. *Cancer*, **37**, 1891–1900.

THIBODEAU SN, BREN G AND SCHAID D. (1993). Microsatellite instability in cancer of the proximal colon. *Science*, **260**, 816–819.

TULCHIN N, ORNSTEIN L, HARPAZ N, GUILLEM J, BORNER C AND O'TOOLE K. (1992). c-myc protein distribution. Neoplastic tissues of the human colon. *Am. J. Pathol.*, **140**, 719–729.

UMAR A, BOYER JC AND KUNKEL TA. (1994). DNA loop repair by human cell extracts. *Science*, **266**, 814–816.

UMPLEBY HC, RANSON DL AND WILLIAMSON RCN. (1985). Peculiarities of mucinous colorectal carcinoma. *Br. J. Surg.*, **72**, 715–718.

VAN DEN BERG F, TIGGES A, SCHIPPER M, DEN HARTOG-JAGER F, KROES W AND WALBOOMERS J. (1989). Expression of the nuclear oncogene p53 in colon tumours. *J. Pathol.*, **157**, 193–199.

VAN DEN INGH HF, RUITER DJ, GRIFFIOEN G, VAN MUIJEN ENP AND FERRONE S. (1987). HLA antigens in colorectal tumours, low expression of HLA class I antigens in mucinous colorectal carcinomas. *Br. J. Cancer*, **55**, 125–130.

VELCICH A AND AUGENLICHT L. (1993). Regulated expression of an intestinal mucin gene in HT29 colonic carcinoma cells. *J. Biol. Chem.*, **268**, 13956–13961.

VOGELSTEIN B, FEARON ER, HAMILTON SR, KERN SE, PREISINGER AC, LEPPERT M, NAKAMURA Y, WHITE R, SMITS AMM AND BOS JL. (1988). Genetic alterations during colorectal tumor development. *N. Engl. J. Med.*, **319**, 525–532.

WATERHOUSE J, MUIR C, SHANMUGARATHAN K AND POWELL J. (1982). *Cancer Incidence in Five Continents*. IARC publication No. 42. IARC: Lyon.

WERNESS BA, LEVINE AJ AND HOWLEY PM. (1990). The E6 proteins encoded by human papillomavirus types 16 and 18 can complex p53 *in vitro*. *Science*, **248**, 76–79.

WILLIAMS A, BROWNE S, YEUDAL W, PATERSON I, MARSHALL C, LANE D AND PARASKEVA C. (1993). Molecular events including p53 and k-*ras* alterations in the *in vitro* progression of a human colorectal adenoma cell line to an adenocarcinoma. *Oncogene*, **8**, 3063–3072.

YAMAMOTO S, MOCHIZUKI H, HASE K, YAMAMOTO T, OHKUSA Y, YOKOYAMA S, USHITANI Y AND TAMAKUMA S. (1993). Assessment of clinicopathologic features of colorectal mucinous adenocarcinoma. *Am. J. Surg.*, **166**, 257–261.

ZAMBETTI GP AND LEVINE AJ. (1993). A comparison of the biological activities of wild-type and mutant p53. *FASEB J.*, **7**, 855–865.

ZHANG SY, RUGGERI B, AGARWAL P, SORLING AP, OBARA T, URA H, NAMIKI M AND KLEIN-SZANTO AJ. (1994). Immunohistochemical analysis of p53 expression in human pancreatic carcinomas. *Arch. Pathol. Lab. Med.*, **118**, 150–154.